# 6-Methyl-4-oxo-4*H*-chromene-3-carbaldehyde

**DOI:** 10.1107/S1600536812037555

**Published:** 2012-09-12

**Authors:** Sammer Yousuf, Asma Mukhtar, Nida Ambreen, Syed Muhammad Saad, Khalid M. Khan

**Affiliations:** a H.E.J. Research Institute of Chemistry, International Center for Chemical and Biological Sciences, University of Karachi, Karachi 75270, Pakistan

## Abstract

In the title compound, C_11_H_8_O_3_, the benzopyran-4-one or chromone ring system is almost planar, with a maximum deviation of 0.045 (2) Å. The crystal structure is stablized by π–π inter­actions between the benzene and pyran rings of inversion-related mol­ecules stacked along the *b* axis, with a centroid–centroid distance of 3.5463 (12) Å

## Related literature

For the biological activity of chromone, see: Patel *et al.* (2011 ▶); Khan *et al.* (2009 ▶, 2010 ▶); Gautam *et al.* (2010 ▶); Ishar *et al.* (2006 ▶); Hassan (1992 ▶); Nohara *et al.* (1974 ▶). For a related structure, see: Wang & Kong (2007 ▶).
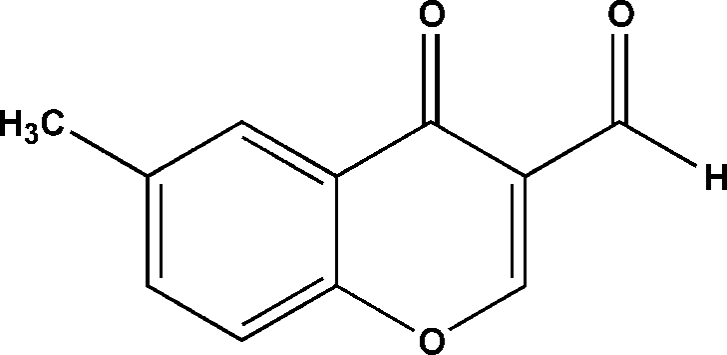


## Experimental

### 

#### Crystal data

C_11_H_8_O_3_
*M* *_r_* = 188.17
Triclinic, 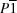

*a* = 6.6945 (7) Å
*b* = 7.1079 (7) Å
*c* = 10.3032 (11) Å
α = 71.593 (2)°
β = 84.962 (2)°
γ = 69.843 (2)°
*V* = 436.57 (8) Å^3^
*Z* = 2
Mo *K*α radiation
μ = 0.11 mm^−1^
*T* = 273 K
0.26 × 0.23 × 0.11 mm

#### Data collection

Bruker SMART APEX CCD area-detector diffractometer
Absorption correction: multi-scan (*SADABS*; Bruker, 2000 ▶) *T*_min_ = 0.973, *T*_max_ = 0.989
4974 measured reflections
1629 independent reflections
1300 reflections with *I* > 2σ(*I*)
*R*_int_ = 0.019

#### Refinement

*R*[*F* ^2^ > 2σ(*F* ^2^)] = 0.049
*wR*(*F* ^2^) = 0.152
*S* = 1.07
1629 reflections
128 parameters
H-atom parameters constrained
Δρ_max_ = 0.26 e Å^−3^
Δρ_min_ = −0.19 e Å^−3^

### 

Data collection: *SMART* (Bruker, 2000 ▶); cell refinement: *SAINT* (Bruker, 2000 ▶); data reduction: *SAINT*; program(s) used to solve structure: *SHELXS97* (Sheldrick, 2008 ▶); program(s) used to refine structure: *SHELXL97* (Sheldrick, 2008 ▶); molecular graphics: *SHELXTL* (Sheldrick, 2008 ▶); software used to prepare material for publication: *SHELXTL*, *PARST* (Nardelli, 1995 ▶) and *PLATON* (Spek, 2009 ▶).

## Supplementary Material

Crystal structure: contains datablock(s) global, I. DOI: 10.1107/S1600536812037555/rz2798sup1.cif

Structure factors: contains datablock(s) I. DOI: 10.1107/S1600536812037555/rz2798Isup2.hkl

Supplementary material file. DOI: 10.1107/S1600536812037555/rz2798Isup3.cml

Additional supplementary materials:  crystallographic information; 3D view; checkCIF report

## Figures and Tables

**Fig. 1.**
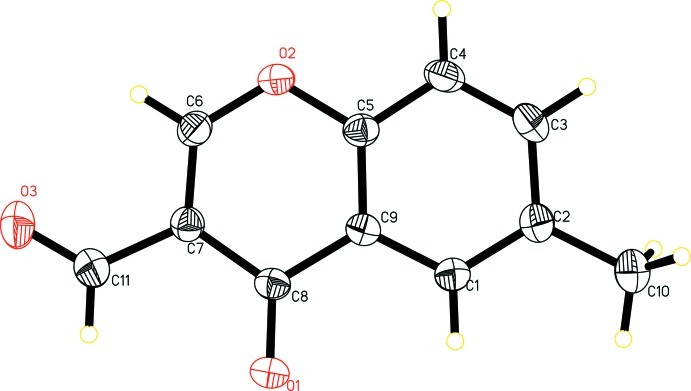
The molecular structure of the title compound with displacement ellipsoids drawn at the 30% probability level.

**Fig. 2.**
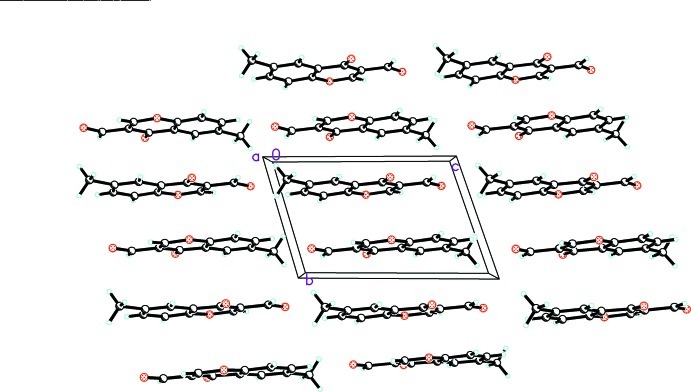
The crystal packing of the title compound viewed along the a axis.

## supplementary crystallographic information

### Comment

Chromone is a heterocyclic compound containing a benzene ring fused with a pyran ring, so it is also called as benzopyran-4-one. Chromone moieties are associated with various physiological and biological properties such as antibacterial (Patel *et al.*, 2011), antioxidant (Gautam *et al.*, 2010; Hassan *et al.*, 1992), antianaphylactic (Nohara *et al.*, 1974), antiinflammatory (Khan *et al.*, 2010), anticancer (Ishar *et al.*, 2006), and thymidine phosphorylase inhibitor (Khan *et al.*, 2009) activities. The title compound is a chromone derivative synthesized as a part of our ongoing research to study different biological activities of this medicinally important class of organic compounds and establish their structure–activity relationship.

The structure of title compound (Fig. 1) is composed of a planar chromone moiety (O1/C1–C9) with maximum deviation of 0.045 (2) Å for atom C8. Bond lengths and angles are similar to those observed in a structurally related compound (Wang & Kong, 2007). In the crystal (Fig. 2), inversion-related molecules are linked along the *b* axis by significant π–π stacking interactions occurring between benzene and pyran rings of chromone moeities, with centroid-centroid distances of 3.5463 (12) Å.

### Experimental

The title compound was synthesized by taking dry dimethylformamide (12.32 ml) into a three necked flask followed by slow addition of POCl_3_ (49 mmol) with intensive stirring at 50°C. Heating and stirring was continued for 2 h at 45–55°C. A solution of 5-methyl-2-hydroxyacetophenone (10 mmol) in DMF was then slowly added under stirring at 50°C. The stirring was continued for additional 2 h at 55–60°C. After cooling, the mixture was kept over night at room temperature and diluted slowly by adding crushed ice (300 g) and stirred again for 6 h to obtain the crude product. Recrystallization from ethanol afforded crystals in 78.7% yield (1.48 g) which were found suitable for single-crystal X-ray diffraction studies. All chemicals were purchased by sigma Aldrich Germany.

### Refinement

H atoms were positioned geometrically with C—H = 0.93–0.95 Å and constrained to ride on their parent atoms with *U*_iso_(H)= 1.5*U*_eq_(CH_3_) or 1.2*U*_eq_(CH).

### Crystal data

**Table:**

C_11_H_8_O_3_	*Z* = 2
*M**_r_* = 188.17	*F*(000) = 196
Triclinic, *P*1	*D*_x_ = 1.431 Mg m^−^^3^
Hall symbol: -P 1	Mo *K*α radiation, λ = 0.71073 Å
*a* = 6.6945 (7) Å	Cell parameters from 1636 reflections
*b* = 7.1079 (7) Å	θ = 3.2–28.1°
*c* = 10.3032 (11) Å	µ = 0.11 mm^−^^1^
α = 71.593 (2)°	*T* = 273 K
β = 84.962 (2)°	Block, colorles
γ = 69.843 (2)°	0.26 × 0.23 × 0.11 mm
*V* = 436.57 (8) Å^3^	

### Data collection

**Table:**

Bruker SMART APEX CCD area-detector diffractometer	1629 independent reflections
Radiation source: fine-focus sealed tube	1300 reflections with *I* > 2σ(*I*)
Graphite monochromator	*R*_int_ = 0.019
ω scan	θ_max_ = 25.5°, θ_min_ = 2.1°
Absorption correction: multi-scan (*SADABS*; Bruker, 2000)	*h* = −8→8
*T*_min_ = 0.973, *T*_max_ = 0.989	*k* = −8→8
4974 measured reflections	*l* = −12→12

### Refinement

**Table:**

Refinement on *F*^2^	Secondary atom site location: difference Fourier map
Least-squares matrix: full	Hydrogen site location: inferred from neighbouring sites
*R*[*F*^2^ > 2σ(*F*^2^)] = 0.049	H-atom parameters constrained
*wR*(*F*^2^) = 0.152	*w* = 1/[σ^2^(*F*_o_^2^) + (0.0852*P*)^2^ + 0.0886*P*] where *P* = (*F*_o_^2^ + 2*F*_c_^2^)/3
*S* = 1.07	(Δ/σ)_max_ < 0.001
1629 reflections	Δρ_max_ = 0.26 e Å^−^^3^
128 parameters	Δρ_min_ = −0.19 e Å^−^^3^
0 restraints	Extinction correction: *SHELXTL* (Sheldrick, 2008), Fc^*^=kFc[1+0.001xFc^2^λ^3^/sin(2θ)]^-1/4^
Primary atom site location: structure-invariant direct methods	Extinction coefficient: 0.013 (9)

### Special details

**Table:**

Geometry. All e.s.d.'s (except the e.s.d. in the dihedral angle between two l.s. planes) are estimated using the full covariance matrix. The cell e.s.d.'s are taken into account individually in the estimation of e.s.d.'s in distances, angles and torsion angles; correlations between e.s.d.'s in cell parameters are only used when they are defined by crystal symmetry. An approximate (isotropic) treatment of cell e.s.d.'s is used for estimating e.s.d.'s involving l.s. planes.
Refinement. Refinement of *F*^2^ against ALL reflections. The weighted *R*-factor *wR* and goodness of fit *S* are based on *F*^2^, conventional *R*-factors *R* are based on *F*, with *F* set to zero for negative *F*^2^. The threshold expression of *F*^2^ > σ(*F*^2^) is used only for calculating *R*-factors(gt) *etc*. and is not relevant to the choice of reflections for refinement. *R*-factors based on *F*^2^ are statistically about twice as large as those based on *F*, and *R*- factors based on ALL data will be even larger.

### Fractional atomic coordinates and isotropic or equivalent isotropic displacement parameters (Å^2^)

**Table:**

	*x*	*y*	*z*	*U*_iso_*/*U*_eq_	
O1	0.2834 (2)	0.1857 (2)	0.62203 (13)	0.0573 (4)	
O2	−0.30931 (18)	0.3149 (2)	0.47149 (12)	0.0476 (4)	
O3	−0.2182 (3)	0.2434 (3)	0.87960 (15)	0.0749 (5)	
C1	0.2333 (3)	0.2150 (3)	0.34035 (18)	0.0415 (4)	
H1A	0.3724	0.1795	0.3703	0.050*	
C2	0.1952 (3)	0.2394 (3)	0.20506 (19)	0.0455 (5)	
C3	−0.0162 (3)	0.2971 (3)	0.16259 (19)	0.0497 (5)	
H3A	−0.0452	0.3169	0.0717	0.060*	
C4	−0.1816 (3)	0.3252 (3)	0.25048 (19)	0.0494 (5)	
H4A	−0.3210	0.3642	0.2199	0.059*	
C5	−0.1371 (3)	0.2942 (3)	0.38609 (18)	0.0397 (4)	
C6	−0.2734 (3)	0.2855 (3)	0.60304 (19)	0.0443 (5)	
H6A	−0.3894	0.2953	0.6604	0.053*	
C7	−0.0835 (3)	0.2431 (3)	0.65872 (18)	0.0396 (4)	
C8	0.1067 (3)	0.2197 (3)	0.57661 (18)	0.0386 (4)	
C9	0.0686 (3)	0.2422 (2)	0.43295 (17)	0.0360 (4)	
C10	0.3734 (4)	0.2037 (4)	0.1063 (2)	0.0654 (6)	
H10A	0.5069	0.1645	0.1524	0.098*	
H10B	0.3537	0.3308	0.0316	0.098*	
H10C	0.3733	0.0931	0.0717	0.098*	
C11	−0.0697 (3)	0.2176 (3)	0.8057 (2)	0.0548 (5)	
H11A	0.0649	0.1784	0.8440	0.066*	

### Atomic displacement parameters (Å^2^)

**Table:**

	*U*^11^	*U*^22^	*U*^33^	*U*^12^	*U*^13^	*U*^23^
O1	0.0400 (8)	0.0876 (10)	0.0504 (8)	−0.0238 (7)	−0.0042 (6)	−0.0252 (7)
O2	0.0330 (7)	0.0611 (8)	0.0441 (7)	−0.0130 (6)	−0.0026 (5)	−0.0121 (6)
O3	0.0733 (11)	0.1117 (13)	0.0537 (9)	−0.0380 (10)	0.0201 (8)	−0.0413 (9)
C1	0.0385 (9)	0.0444 (10)	0.0434 (10)	−0.0157 (8)	0.0003 (7)	−0.0138 (8)
C2	0.0522 (11)	0.0442 (10)	0.0401 (10)	−0.0176 (8)	0.0025 (8)	−0.0117 (8)
C3	0.0609 (12)	0.0530 (11)	0.0348 (9)	−0.0209 (9)	−0.0073 (8)	−0.0087 (8)
C4	0.0436 (10)	0.0553 (11)	0.0459 (11)	−0.0163 (9)	−0.0118 (8)	−0.0077 (8)
C5	0.0373 (9)	0.0369 (9)	0.0423 (10)	−0.0120 (7)	−0.0017 (7)	−0.0085 (7)
C6	0.0389 (10)	0.0465 (10)	0.0440 (10)	−0.0114 (8)	0.0043 (7)	−0.0133 (8)
C7	0.0419 (10)	0.0387 (9)	0.0403 (10)	−0.0145 (7)	0.0017 (8)	−0.0141 (7)
C8	0.0369 (9)	0.0385 (9)	0.0427 (9)	−0.0131 (7)	−0.0033 (7)	−0.0137 (7)
C9	0.0372 (9)	0.0325 (8)	0.0384 (9)	−0.0124 (7)	−0.0024 (7)	−0.0094 (7)
C10	0.0634 (14)	0.0885 (16)	0.0457 (11)	−0.0245 (12)	0.0091 (10)	−0.0256 (11)
C11	0.0550 (12)	0.0696 (13)	0.0482 (11)	−0.0241 (10)	0.0037 (9)	−0.0262 (10)

### Geometric parameters (Å, º)

**Table:**

O1—C8	1.227 (2)	C4—H4A	0.9300
O2—C6	1.335 (2)	C5—C9	1.385 (2)
O2—C5	1.383 (2)	C6—C7	1.339 (3)
O3—C11	1.196 (2)	C6—H6A	0.9300
C1—C2	1.385 (3)	C7—C8	1.455 (2)
C1—C9	1.396 (2)	C7—C11	1.475 (3)
C1—H1A	0.9300	C8—C9	1.473 (2)
C2—C3	1.399 (3)	C10—H10A	0.9600
C2—C10	1.505 (3)	C10—H10B	0.9600
C3—C4	1.368 (3)	C10—H10C	0.9600
C3—H3A	0.9300	C11—H11A	0.9300
C4—C5	1.387 (3)		
			
C6—O2—C5	117.99 (13)	C6—C7—C8	120.85 (16)
C2—C1—C9	121.75 (17)	C6—C7—C11	118.72 (16)
C2—C1—H1A	119.1	C8—C7—C11	120.43 (16)
C9—C1—H1A	119.1	O1—C8—C7	123.32 (16)
C1—C2—C3	117.68 (17)	O1—C8—C9	122.63 (16)
C1—C2—C10	121.76 (18)	C7—C8—C9	114.04 (15)
C3—C2—C10	120.56 (17)	C5—C9—C1	118.08 (16)
C4—C3—C2	122.15 (17)	C5—C9—C8	119.66 (16)
C4—C3—H3A	118.9	C1—C9—C8	122.26 (16)
C2—C3—H3A	118.9	C2—C10—H10A	109.5
C3—C4—C5	118.60 (17)	C2—C10—H10B	109.5
C3—C4—H4A	120.7	H10A—C10—H10B	109.5
C5—C4—H4A	120.7	C2—C10—H10C	109.5
O2—C5—C9	122.29 (16)	H10A—C10—H10C	109.5
O2—C5—C4	116.01 (15)	H10B—C10—H10C	109.5
C9—C5—C4	121.69 (16)	O3—C11—C7	125.06 (19)
O2—C6—C7	125.04 (16)	O3—C11—H11A	117.5
O2—C6—H6A	117.5	C7—C11—H11A	117.5
C7—C6—H6A	117.5		
			
C9—C1—C2—C3	1.3 (3)	C6—C7—C8—C9	−1.3 (2)
C9—C1—C2—C10	−178.14 (16)	C11—C7—C8—C9	177.95 (15)
C1—C2—C3—C4	−1.3 (3)	O2—C5—C9—C1	176.96 (14)
C10—C2—C3—C4	178.17 (18)	C4—C5—C9—C1	−1.9 (3)
C2—C3—C4—C5	−0.3 (3)	O2—C5—C9—C8	−3.7 (3)
C6—O2—C5—C9	0.8 (2)	C4—C5—C9—C8	177.44 (15)
C6—O2—C5—C4	179.73 (15)	C2—C1—C9—C5	0.3 (3)
C3—C4—C5—O2	−176.99 (15)	C2—C1—C9—C8	−179.10 (15)
C3—C4—C5—C9	2.0 (3)	O1—C8—C9—C5	−175.79 (16)
C5—O2—C6—C7	2.0 (3)	C7—C8—C9—C5	3.7 (2)
O2—C6—C7—C8	−1.6 (3)	O1—C8—C9—C1	3.6 (3)
O2—C6—C7—C11	179.14 (16)	C7—C8—C9—C1	−176.89 (14)
C6—C7—C8—O1	178.25 (17)	C6—C7—C11—O3	−4.4 (3)
C11—C7—C8—O1	−2.5 (3)	C8—C7—C11—O3	176.35 (19)
